# Mechanisms predictive of Tibetan Medicine *Sophora moorcroftiana* alkaloids for treatment of lung cancer based on the network pharmacology and molecular docking

**DOI:** 10.1186/s12906-024-04342-3

**Published:** 2024-01-20

**Authors:** Peng Ji, Nian-Shou Zhao, Fan-Lin Wu, Yan-Ming Wei, Ci-Dan Laba, Cuo-Mu Wujin, Yong-Li Hua, Zi-Wen Yuan, Wan-Ling Yao

**Affiliations:** 1 College of Veterinary Medicine of Gansu Agricultural University, Lanzhou, 730070 Gansu China; 2 Institute of Animal Sciences, Tibet Academy of Agricultural Sciences, Tibet Lhasa, 850009 China

**Keywords:** *Sophora Moorcroftiana*, Alkaloids, Lung cancer, Network pharmacology, Molecular docking, Vitro Verification

## Abstract

**Background:**

Leguminous *Sophora moorcroftiana* (SM) is a genuine medicinal material in Tibet. Many research results have reveal the *Sophora moorcroftiana* alkaloids (SMA), as the main active substance, have a wide range of effects, such as antibacterial, antitumor and antiparasitic effects. However, there are few reports on the inhibition of lung cancer (LC) and its inhibitory mechanism, and the pharmacological mechanism of SMA is still unclear, Therefore, exploring its mechanism of action is of great significance.

**Methods:**

The SMA active components were obtained from the literature database. Whereas the corresponding targets were screened from the PubChem and PharmMapper database, UniProt database were conducted the correction and transformation of UniProt ID on the obtained targets. The GeneCards and OMIM databases identified targets associated with LC. Venny tools obtained the intersection targets of SMA and LC. R language and Cytoscape software constructed the visual of SMA - intersection targets – LC disease network. The intersection targets protein-protein interaction (PPI) network were built by the STRING database. The functions and pathways of the common targets of SMA and LC were enriched by gene ontology (GO) and Kyoto Encyclopedia of Genes and Genomes (KEGG). Subsequently, molecular docking And A549 cells vitro experiment were performed to further validate our finding.

**Results:**

We obtained six kinds of alkaloids in SM, 635 potential targets for these compounds, and 1,303 genes related to LC. SMA and LC intersection targets was 33, including ALB, CCND1, ESR1, NOTCH1 and AR. GO enrichment indicated that biological process of SMA was mainly involved in the positive regulation of transcription and nitric oxide biosynthetic process, and DNA-templated, etc. Biological functions were mainly involved in transcription factor binding and enzyme binding, etc. Cell components were mainly involved in protein complexes, extracellular exosome, cytoplasm and nuclear chromatin, etc., Which may be associated with its anti-LC effects. KEGG enrichment analysis showed that main pathways involved in the anti-LC effects of SMA, including pathway in cancer, non small-cell lung cancer, p53, PI3K-Akt and FOXO signaling pathways. Molecular docking analyses revealed that the six active compounds had a good binding activity with the main therapeutic targets 2W96, 2CCH and 1O96. Experiments in vitro proved that SMA inhibited the proliferation of LC A549 cells.

**Conclusions:**

Results of the present study, we have successfully revealed the SMA compounds had a multi-target and multi-channel regulatory mechanism in treatment LC, These findings provided a solid theoretical reference of SMA in the clinical treatment of LC.

## Introduction

Lung cancer (LC), as an exceedingly significant public health problem in developing and developed countries, has a high incidence and mortality rate, and it is one of the most common malignant tumors in clinical practice [1]. In recent years, the incidence and mortality rate of LC patients have been showing an upward trend. Currently, although the surgery treatment for LC can be effective, the overall prognosis is poor [2]. With its advantages of low toxicity, and multi-component and multi-target mechanism [3], traditional Chinese medicine can not only provide new and effective candidate drugs for the treatment of cancer diseases, but also has a good application prospect and research value [4]. The combination of traditional Chinese and Western medicine can effectively treat LC and significantly improve the efficiency of treatment [5]. Therefore, screening the effective components of traditional Chinese medicine as effective drugs for treating LC is of great significance.

Leguminous *Sophora moorcroftiana* (SM) is a genuine medicinal material in Tibet. Its alkaloids have various biological activities, including bacteriostasis, antitumor, and anti-parasite [6]. A continuous in-depth study on the biological activity of SMA found that the inhibitory effect of alkaloid extracts on the proliferation of lung adenocarcinoma cells SPC-A-1 and GLC-82 showed significant dose and time effects. Still, the mechanism of action was unclear [7].

As a new analytical technology, network pharmacology analysis can integrate biological multicomponent and multitarget activities, because it is consistent with the integrity and systematicness of traditional Chinese medicine [8–10]. This discipline is suitable for the in-depth mining of potential active ingredients in traditional Chinese medicine, especially for screening and exploring the mechanism of the effective components of a single drug [11, 12]. Molecular docking technology can predict the binding mode and binding energy of the receptor and ligand by knowing the structural characteristics of the receptor and ligand and the interaction force between them, and further screen out the optimal binding mode of drug molecular ligands and docking receptors. This technology can not only reveal the molecular mechanism of receptor-ligand binding, but also contribute to the pharmacology of traditional Chinese medicine compounds and single drugs. Therefore, this study aim was to apply network pharmacology, molecular docking and cell Experimental Verification to reveal the underlying mechanism by which SMA effects for LC, and to predict the main active compounds and core targets of SMA for the treatment of LC, as well as provide a scientific basis for the biological activity interpretation of LC.

## Materials and methods

### Potential active ingredients target and gene name tagging of SMA components

‘*Sophora Moorcroftiana*’ was used as a search term in CNKI literature database (https://www.cnki.net/) and Chemical constituents confirmed by NMR and MS were screened out from the literature [13, 14]. All the chemical components and structural formulas were obtained from the ChemDraw 17.1.0 software and the use of the Search option ‘Find structure from name at ChemACX. Com’. PubChem database (https://pubchem.ncbi.nlm.nih.gov) was used to search (3D) chemical structure of SMA, the 3D structure was saved as an SDF file and imported into PharmMapper database (http://www.lilab-ecust.cn/pharmmapper) for prediction, with the use of the keywords ‘druggable pharmaceutical models’ to acquire the potential targets of chemical components, the UniProt ID was corrected and transformed by using the UniProt database (https://www.uniprot.org/uniport).

### Prediction of target genes for LC

The search term ‘Lung cancer’ was used to search the GeneCards database (https://www.genecards.org/) and OMIM database (https://omim.org/), GeneCards database results were screened with correlation score > 20. OMIM database results were screened with ‘gene Map’ option, finally, the two database valuable targets were merged, and the repeated targets were deleted to screen LC-related potential valuable targets.

### Construction of PPI network and Screening the therapeutic intersection target for LC

In this study, venny 2.1.0 online tool (https://bioinfogp.cnb.csic.es/tools/venny/) combined with R 3.6.3 software was used to construct the visualization of drug components, disease targets and intersection targets. The interaction regulatory network of the drug components and disease target was constructed by using Cytoscape 3.7.0 software. The interaction regulatory network of the intersection targets was constructed by using the STRING database (https://string-db.org/). The intersection targets were selected from the PPI network.

### GO and KEGG pathway enrichment analysis

To further explore the mechanisms of SMA for LC treatment, the intersection targets was input into the DAVID database (https://david.ncifcrf.gov/), and the selected identifier was set as ‘official gene symbol’. The Biological Process (BP), Cell Component (CC), Molecular Function (MF) and Kyoto Encyclopedia of Genes and Genomes (KEGG) pathway were obtained and analyzed. Finally, the GO functions and KEGG pathway were visualized by using R 3.6.3 software.

### Molecular docking

The intersection targets proteins of LC, which is the active component of SMA, was ligated. The specific operation includes the following aspects: (1) The structure of SMA active components was constructed by using ChemOffice 2017 software based on ChemDraw Professional, and the energy of the obtained structure was minimized in Chem 3D; (2) The 3D structures of the core target protein was obtained from the PDB database (http://www.rcsb.org/); (3) To separate the original ligand and remove the hydrone and other inactive ligands from the core target protein by using PyMol software; (4) Autodock was used to process and dock the protein receptors and molecular ligands. Pymol was used to visualize the docking results.

### Cell experiment verification

#### Obtaining SMA extract

In the early experiment, the extraction scheme of SMA was optimized. The extraction was carried out under 75% ethanol concentration, ultrasonic temperature 55℃, solid–liquid ratio 1 g/mL: 30 g/mL, ultrasonic power 150 W and ultrasonic time 50 min. After the extract is filtered and concentrated in a rotary evaporator, it is dried in a vacuum drying oven to obtain the dry powder of the section of SM.

### Cell culture and proliferation assay

LC A549 cells were obtained from the Lanzhou General Hospital of Lanzhou Military Region. LC A549 cells in logarithmic growth stage were taken and their concentration was adjusted to 2 × 10^5^ pieces/mL, 100 µL per well, inoculated on 96 well plates, cultured at 80% confluence, 5% carbon dioxide and 37℃ cultured 24 h. Then add the solution of SMA at the concentrations of 3000, 1500, 750 and 375 µg/mL, six multiple wells at each concentration, and then treated for 12, 24 and 36 h. After cells were incubated with CCK-8, and the optical density (OD) was measured at the absorption wavelength of 450 nm. According to the formula: cell proliferation inhibition rate = (control hole—experimental hole) / (control hole—blank hole) × The inhibition rate of cells was calculated at 100%.

### Statistical analysis

All data were expressed as the mean ± standard deviation, and the measurement results were analyzed with SPSS 26.0 statistical software performed one-way ANOVA. Statistical significance was defined as P-values, P ≤ 0.05 was considered statistically significant, *P* ≤ 0.01 was considered statistically have extremely significant.

## Results

### Drug ingredients of SMA and obtaining the SMA component- LC disease potential targets

**Fig. 1:**
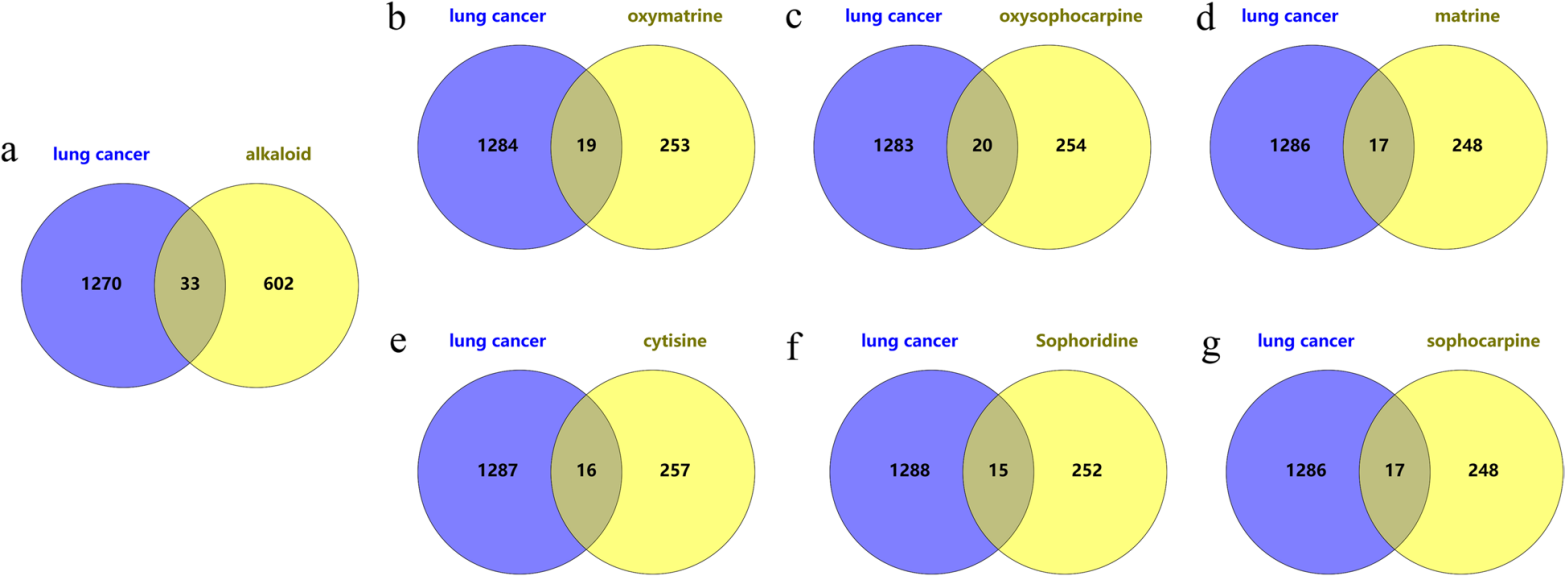
The intersection target of SMA and LC. The blue represents LC disease target, and yellow represents alkaloid target. **a** The Intersection targets of SMA and LC potential targets. **b**-**g** The Intersection targets of oxymatrine, oxysophocarpine, matrine, cytisine, sophoridine, sophocarpine and LC potential targets

According to the collected literature, matrine, oxymatrine, sophocarpine, oxysophocarpine, sophoridine and cytisine component were obtained [9, 10]. The selected six component was confirmed by NMR and MS. The SMA component targets obtained from the PharmMapper database were corrected and transformed by using the UniProt database. Repeats and false positives were removed. A total of 635 potential targets were obtained, and 1303 LC targets were obtained from the GeneCards and OMIM database. There are a total of thirty-three intersection targets between SMA and LC. LC shares targets with oxymatrine, oxysophocarpine, matrine, cytisine, sophoridine and sophocarpine alkaloids accounting for 1.2%, 1.3%, 1.1%, 1%, 1% and 1.1% respectively. Among them, oxysophocarpine has the highest number of intersection targets with LC (Fig. 1).

### Construction of SMA component and LC disease intersection targets network

**Fig. 2:**
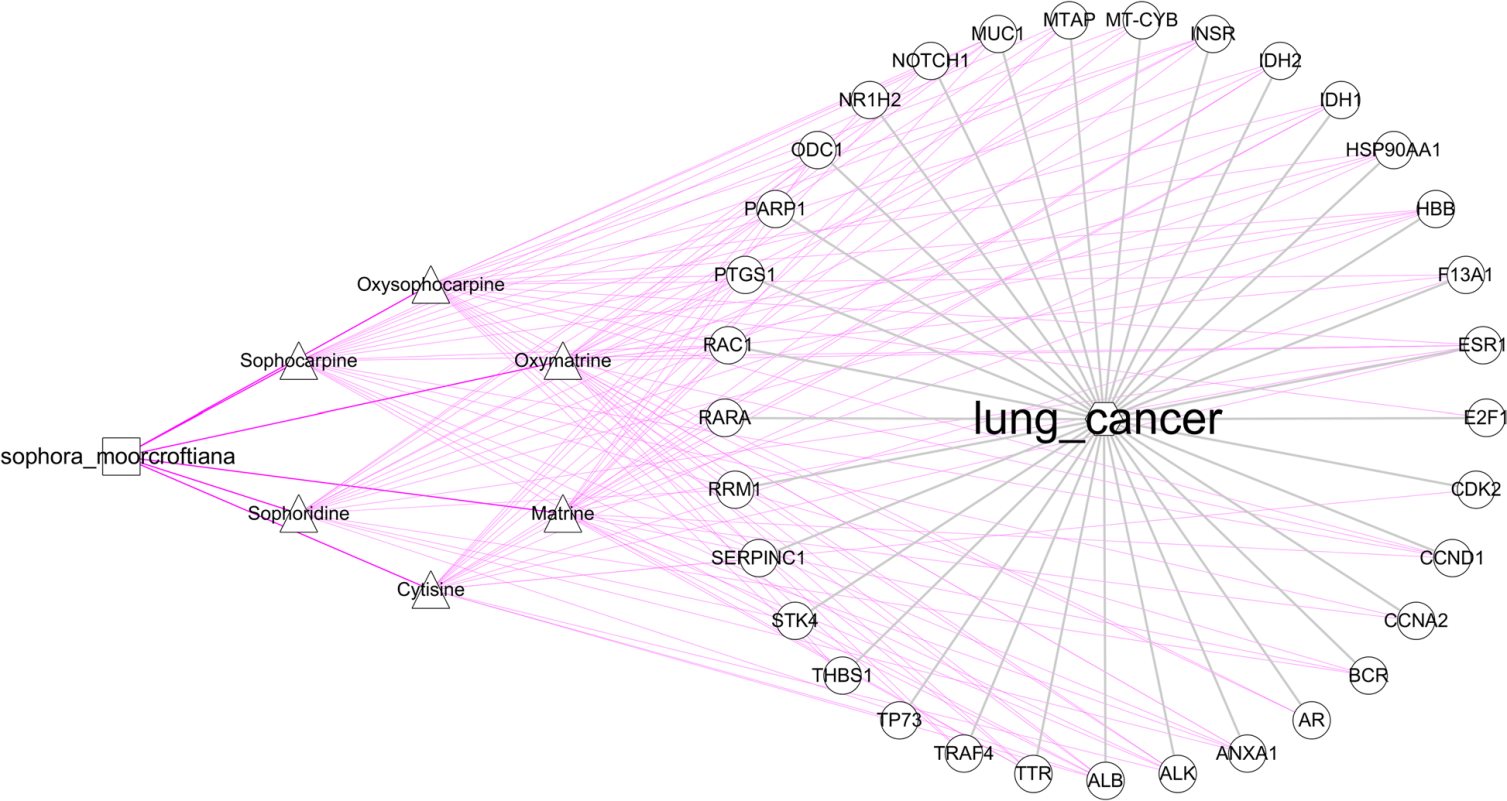
SMA and LC intersection targets network. The square represents SM, the triangle represents the SMA component, the circle represents the intersection targets, and the hexagon represents LC disease

The control network of the drug component target disease was constructed by using Cytoscape 3.7.0. The node represents the SM and SMA component, SMA component and LC disease target, while the edge shows the correlation between the drug component and action targets (Fig. 2).

### Construction of PPI interaction regulatory network of SMA and LC intersection targets

**Fig. 3:**
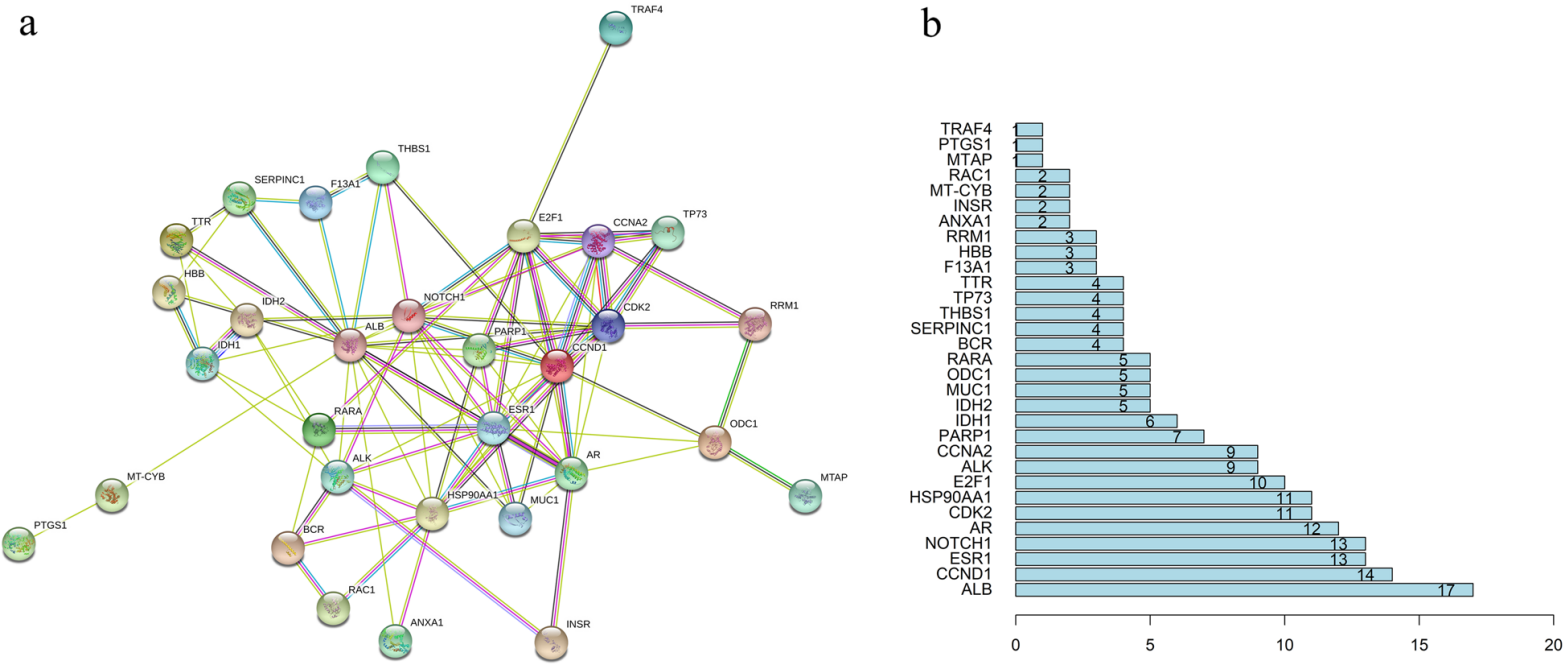
Protein and protein interaction network (PPI) and interaction network node value. **a** Interaction network of target proteins in SMA and LC. **b** Interaction network node value in SMA and LC. The Y-axis shows the target genes. The X-axis shows the gene count

The PPI interaction regulatory network of the intersection target constructed by the STRING database contains 33 nodes and 96 edges. The average node degree value is 5.82, and the local clustering coefficient is 0.53. The nodes in the network represent the targets, the edges between nodes represent protein and protein interactions, and the different colors represent different interactions (Fig. 3a). The targets with higher degree values were ALB (degree: 17), CCND1 (degree: 14), ESR1 (degree: 13), NOTCH1 (degree: 13), AR (degree: 12), CDK2 (degree: 11), HSP90AA1 (degree: 11) (Fig. 3b).

### Go enrichment analysis

**Fig. 4:**
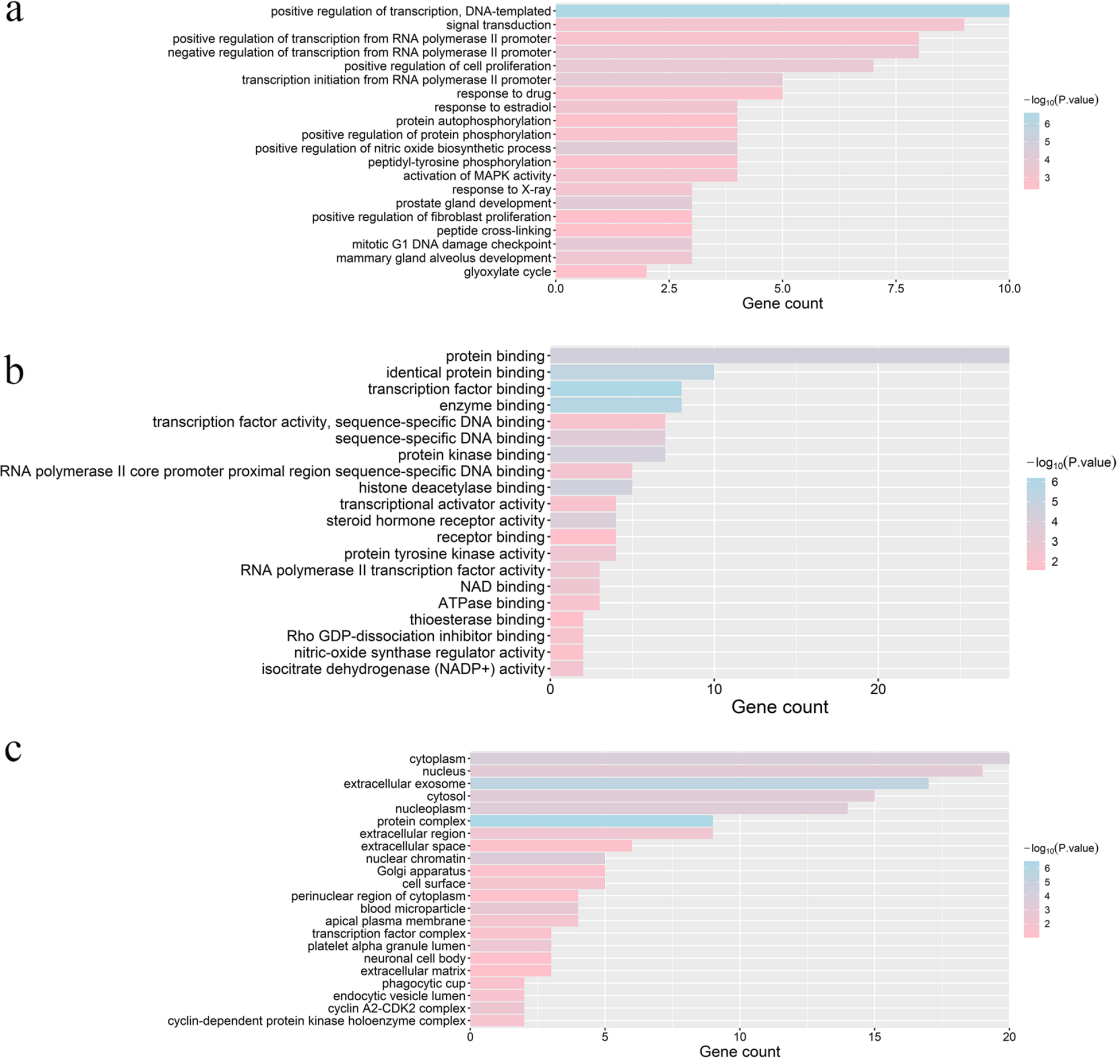
GO enrichment analysis. **a** Biological Process (BP) enriched. **b** Molecular Function (MF) enriched. **c** Cellular Component (CC) enriched. The Y-axis shows the significantly enriched GO categories of the target genes (*p*-value < 0.01), the X-axis indicates significantly enriched BP, MF and CC count of targets

A total of 129 GO entries were enriched in the DAVID databases, 76 of which were BP, 31 were MF, and 22 were CC, accounting for 59%, 24% and 17%, respectively. The most significant BP involves the positive regulation of transcription, DNA-templated (GO: 0045893), positive regulation of nitric oxide biosynthetic process (GO: 0045429) and prostate gland development (GO: 0030850)(Fig. 4a). The most significant MF involves transcription factor binding (GO: 0008134), enzyme binding (GO: 0019899) and identity protein binding (GO: 0042802)(Fig. 4b). The most significant CC involved protein complex (GO: 0043234), extracellular exosomes (GO: 0070062), cytoplasms (GO: 0005737) and nuclear chromatin (GO: 0000790) (Fig. 4c).

### KEGG enrichment analysis

**Fig. 5:**
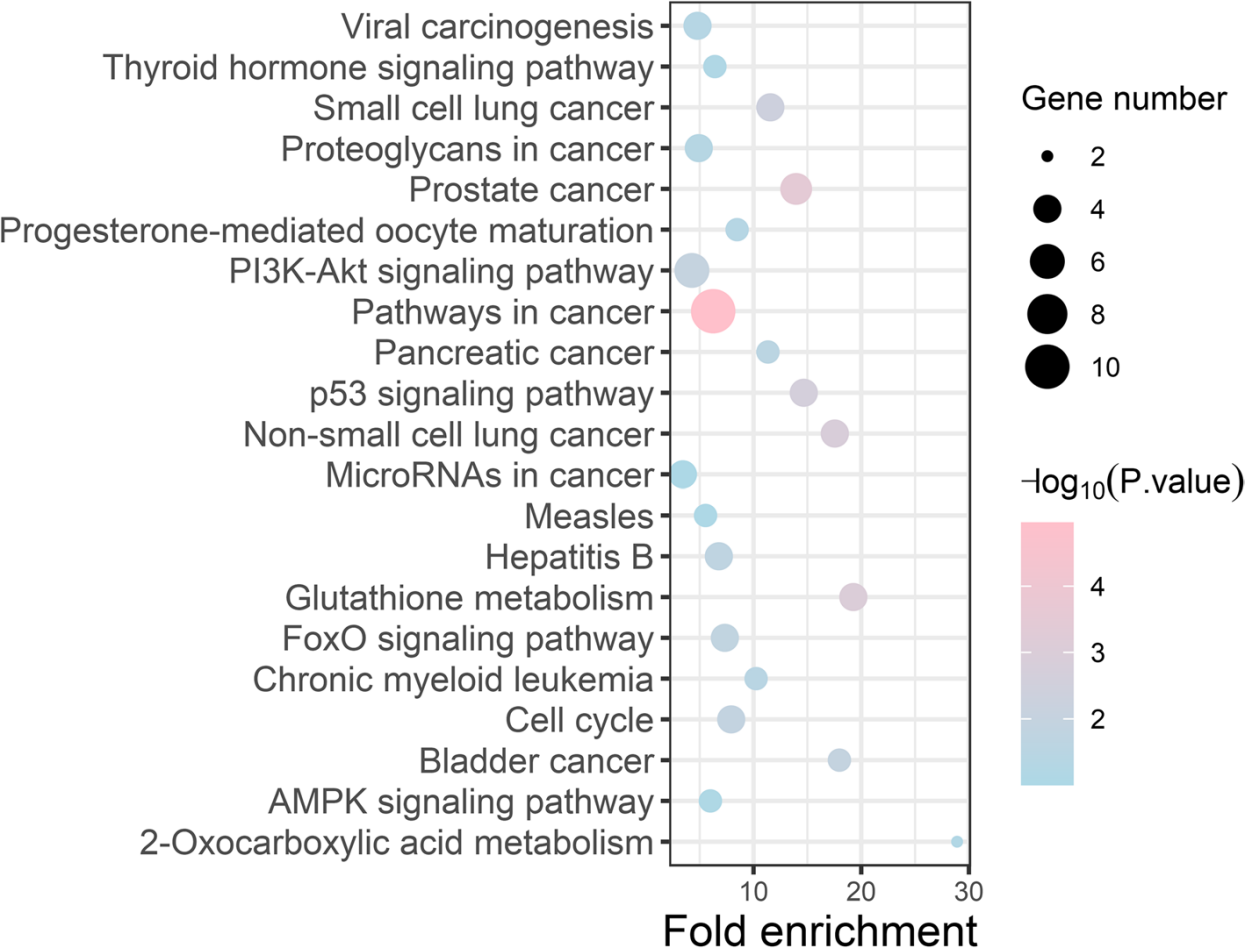
KEGG enrichment pathway. The Y-axis shows enriched KEGG pathways of the target genes, the X-axis displayed the rich factor (*p*-value < 0.01) and the dot's color reflects the different value ranges

Twenty-one KEGG pathways were enriched. The most significant pathways were pathways in cancer (hsa05200) and the PI3K/Akt signaling pathway (hsa04151), followed by the p53 signaling pathway (hsa04115), glutathione metabolism (hsa00480), non-small cell lung cancer (hsa05223) and prostate cancer (hsa05215) (Fig. 5). And the SMA- LC intersection targets KEGG enrichment pathway and gene name (Table 1).

**Table 1:** SMA- LC intersection targets KEGG enrichment pathway and gene name

NO	Term	count	*P*-Value	Genes
1	hsa05200: 21Pathways in cancer	10	9.90 × 10^–4^	E2F1、AR、CCND1、HSP90AA1、BCR、RAC1、RARA、STK4、TRAF4、CDK2
2	hsa05215: Prostate cancer	5	3.50 × 10^–4^	E2F1、AR、CCND1、HSP90AA1、CDK2
3	hsa00480: Glutathione metabolism	4	8.60 × 10^–4^	ODC1、RRM1、IDH2、IDH1
4	hsa05223: Non-small cell lung cancer	4	1.30 × 10^–3^	E2F1、CCND1、ALK、STK4
5	hsa04115: p53 signalling pathway	4	2.50 × 10^–3^	CCND1、THBS1、CDK2、TP73
6	hsa05222: Small cell lung cancer	4	4.90 × 10^–3^	E2F1、CCND1、TRAF4、CDK2
7	hsa04151: PI3K-Akt signaling pathway	6	9.10 × 10^–3^	CCND1、HSP90AA1、RAC1、THBS1、INSR、CDK2
8	hsa05219: Bladder cancer	3	1.10 × 10^–2^	E2F1、CCND1、THBS1
9	hsa04110: Cell cycle	4	1.20 × 10^–2^	E2F1、CCND1、CCNA2、CDK2
10	hsa04068: FoxO signaling pathway	4	1.30 × 10^–2^	CCND1、STK4、INSR、CDK2
11	hsa05161: Hepatitis B	4	2.00 × 10^–2^	E2F1、CCND1、CCNA2、CDK2
12	hsa05212: Pancreatic cancer	3	2.80 × 10^–2^	E2F1、CCND1、RAC1
13	hsa05220: Chronic myeloid leukemia	3	3.40 × 10^–2^	E2F1、CCND1、BCR
14	hsa05205: Proteoglycans in cancer	4	4.20 × 10^–2^	CCND1、RAC1、ESR1、THBS1
15	hsa05203: Viral carcinogenesis	4	4.20 × 10^–2^	CCND1、RAC1、CCNA2、CDK2
16	hsa04914: Progesterone-mediated oocyte maturation	3	4.30 × 10^–2^	HSP90AA1、CCNA2、CDK2
17	hsa01210: 2-Oxocarboxylic acid metabolism	2	6.00 × 10^–2^	IDH2、IDH1
18	hsa04919: Thyroid hormone signaling pathway	3	7.30 × 10^–2^	NOTCH1、CCND1、ESR1
19	hsa04152: AMPK signaling pathway	3	7.90 × 10^–2^	CCND1、CCNA2、INSR
20	hsa05162: Measles	3	9.60 × 10^–2^	CCND1、CDK2、TP73
21	hsa05206: MicroRNAs in cancer	4	8.00 × 10^–2^	E2F1、NOTCH1、CCND1、THBS1

### Molecular docking

**Fig. 6:**
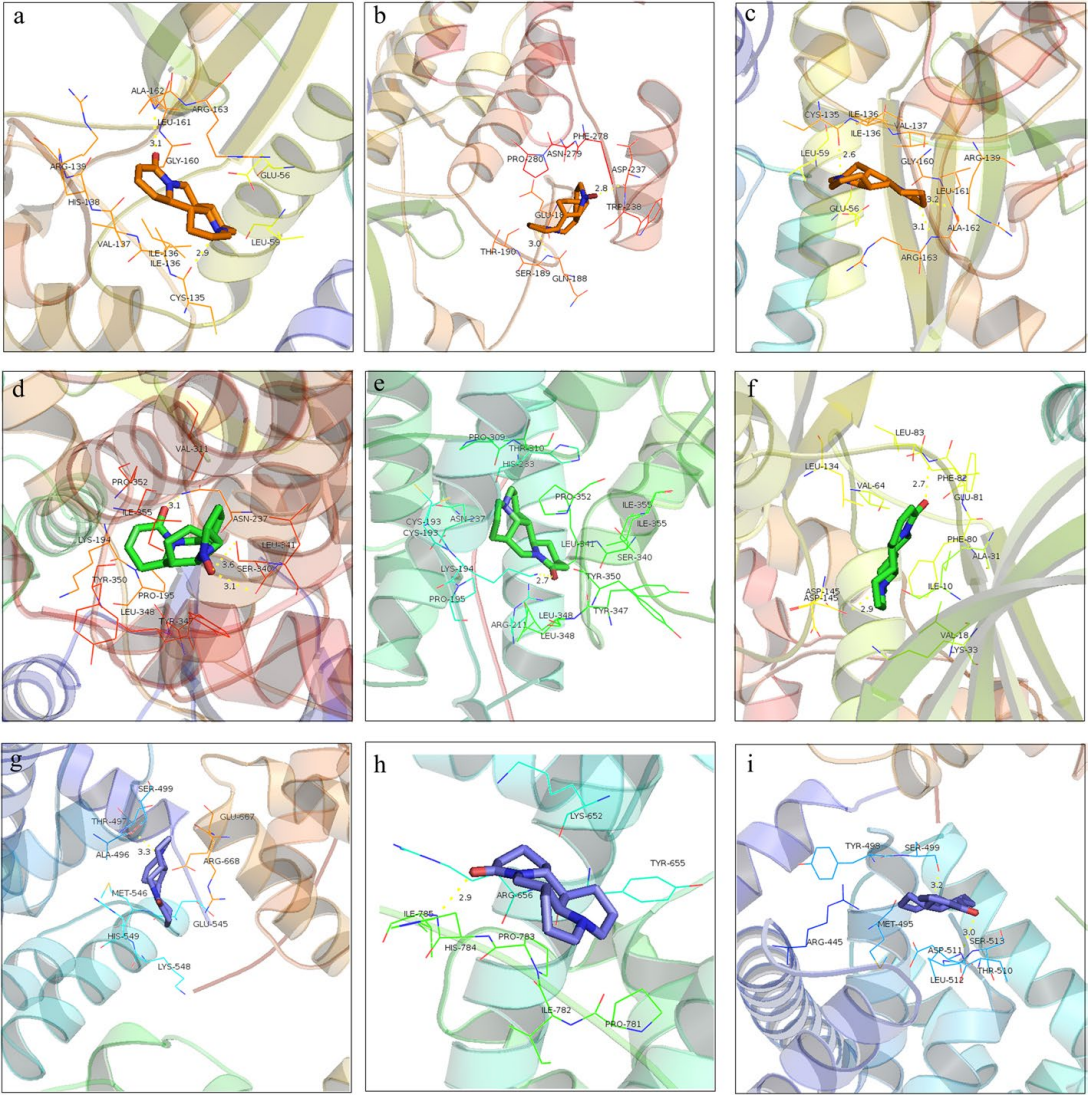
Molecular docking pattern of intersection targets of SMA in LC treatment. **a** Binding pattern of 2W96 protein and sophocarpine. **b** The binding pattern of 2W96 protein and oxysophocarpine. **c** The binding pattern of 2W96 protein and sophoridine. **d** The binding pattern of 2CCH protein and oxysophocarpine. **e** The binding pattern of 2CCH protein and sophoridine. **f** The binding pattern of 2CCH protein and sophocarpine. **g** The binding pattern of 1O96 protein and matrine. **h** The binding pattern of 1O96 protein and sophoridine. **i** The binding pattern of 1O96 protein and sophocarpine

Six alkaloids from SM were linked with CCND1 (2W96), CDK2 (2CCH) and E2F1 (1O96) targets. The docking of each active component and protein produces ten docking results. According to the principle of a lower binding energy corresponding to an increased possibility of interaction between protein and molecule and to a stable binding conformation, the conformation with the lowest binding energy of ligand and receptor is selected as the docking conformation. The results showed the binding energies of alkaloid components and target proteins were all less than -4 kJ/mol, indicating that the energetic drug molecules could bind to the target protein well. the binding sites of monomer components with protein amino acids and the binding length of hydrogen bonds (Table 2 and Fig. 6).

**Table 2:** The binding energy of docking information of intersection targets protein with SMA

NO	Active ingredient	Molecular formula	Structure	Active ingredient	PDB ID	Binding energy(KJ/mol)
1	matrine	C_15_H_24_N_2_O		CCND1	2W96	-7.71
				CDK2	2CCH	-7.5
				E2F1	1O96	-6.52
2	oxymatrine	C_15_H_24_N_2_O_2_		CCND1	2W96	-7.51
				CDK2	2CCH	-7.89
				E2F1	1O96	-4.69
3	sophoridine	C_15_H_24_N_2_O		CCND1	2W96	-7.7
				CDK2	2CCH	-8.18
				E2F1	1O96	-6.23
4	sophocarpine	C_15_H_22_N_2_O		CCND1	2W96	-8.34
				CDK2	2CCH	-8.05
				E2F1	1O96	-6.12
5	oxysophocarpine	C_15_H_22_N_2_O_2_		CCND1	2W96	-7.79
				CDK2	2CCH	-8.2
				E2F1	1O96	-5.0
6	cytisine	C_11_H_14_N_2_O		CCND1	2W96	-6.83
				CDK2	2CCH	-6.47
				E2F1	1O96	-5.87

### *Cell experiment *in vitro

**Fig. 7:**
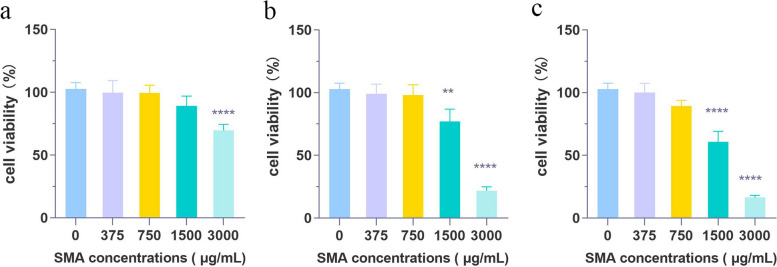
SMA cell viability of A549 cells. **a**-**c** A549 cells were treated in SMA (375, 750, 1500 and 3000 μg/mL), respectively for 12, 24 and 36 h, the viability was measured by CCK8 assay. Data are expressed as the mean ± standard deviation of two replicates. "**"*p* < 0.01, "****"*p* < 0.0001 vs control group

The results showed the alcohol extract of SMA acted on the A549 cell line, the cell proliferation of the cell line was inhibited to varying degrees. The adherent cells were rounded and floated under an inverted microscope, compared with the control group, the OD_450_ of the drug treatment group changed most significantly at 24 and 36 h. As the drug concentration increased from 375 to 3000 µg/mL, the inhibited proliferation was gradually increased, and the inhibited proliferation at 36 h was the highest. The cell inhibition rates of 375, 750, 1500 and 3000 µg/mL after 36 h treatment were 1.29%, 10.84%, 39.55% and 83.69%, respectively, which further shows that the SMA on LC A549 cells are time and dose-dependent (Fig. 7).

## Discussion

In this study, we use network pharmacological approach to reveal the potential moleculai mechanisms of SMA in LC. Through Cytoscape software and PPI network 33 core targets were screened out from the SMA component and LC disease regulatory network. Among the intersection targets of SMA components and LC, oxysophocarpine (degree: 20) has the highest number, followed by oxymatrine (degree: 19). Previous research shows that oxymatrine, matrine, oxysophocarpine, sophocarpine, sophoridine and cytisine have different inhibitory effects on LC cell lines [15–19]. Such as oxymatrine can induce apoptosis of the human LC non-small cell LC cell line A549 [20, 21], and oxysophocarpine has an excellent inhibitory effect on liver cancer, colorectal cancer and other cell lines [22, 23].

By constructing the SMA and LC disease intersection targets gene interaction regulatory network, the targets with degree > 10 were selected as ALB, CCND1, ESR1, Notch1, AR, CDK2, HSP90AA1 and E2F1. CCND1 and CDK2 were both expressed in non-small cell lung cancer, and CCND1 was highly expressed in non-small cell lung cancer tissues (42%). The expression of CDK2 in non-small cell lines and tissue samples was negatively correlated with the expression of microRNA-597 [24]. The rs3213172C/T polymorphism of the E2F1 gene can be used as an effective biomarker for the genetic susceptibility of LC in the Chinese population [25].Therefore, the selected core targets can not only be used as markers of LC but may also become one of the key targets for the treatment of LC.

Based on the GO prediction of the BP pathway, MF pathway, CC pathway and KEGG signaling pathways by DAVID, the biological pathway is involved in the positive regulation of DNA template transcription; the molecular pathway is involved in transcription factor binding, enzyme binding and the same protein binding; and the cellular pathway is involved in protein complexes and exosomes. These results indicated that SMA might affect protein recombination by affecting protein complexes. The protein and exosomes were used to regulate the binding process of enzymes, thus affecting the transcriptional regulation of DNA templates.

Among the 21 pathways, the most significantly enriched pathways include Pathways in cancer, Thyroid hormone signaling pathway, PI3K/Akt signaling pathway, P53 signaling pathway, FoxO signaling pathway and AMPK signaling pathway. The PI3K/Akt pathway is involved in important processes, such as cell proliferation, cell cycle progression, metastasis, survival and apoptosis. It is also one of the most important oncogenic targets in almost all types of cancer. The PI3K/Akt signaling pathway plays a crucial role in the occurrence and metastasis of lung cancer, not only showing high expression in non-small cell lung cancer [26], but also playing a key role in the cell survival and being abnormally activated in the development of cancer. Such as ILTPs inhibit the growth and proliferation of lung cancer A549 cells by regulating the PI3K/Akt signaling pathway, suppressing the PI3K/Akt pathway activated by oxidative stress, and inhibiting the expression of PI3K and Akt [27, 28]. Camellia Leave Saponins can significantly inhibit the proliferation of NCI-H1975, A549 and HCC827 cells, inducing cell autophagy and suppressing tumor growth through the PI3K and MAPK signaling pathways [29]. Isoliquiritigenin, a compound found in licorice, induces growth inhibition and cell apoptosis in A549 lung cancer cells by inhibiting the activation of the PI3K/AKT/mTOR signaling pathway [30]. Docosahexaenoic acid, a 22-carbon omega-3 fatty acid, induces cell death in human non-small cell lung cancer cells by inhibiting mTOR through the PI3K/AKT pathway [31]. White peony extract induces cell apoptosis and autophagic cell death in human lung cancer cells by inhibiting the PI3K/Akt/mTOR pathway [32]. The nuclear translocation of nuclear factor κB (NF-κB) promotes the expression of p65 protein and the transcription of CCND1. The overexpression of CCND1 further promotes the development of lung cancer A549, leading to cell proliferation and inducing cell cycle arrest at the S phase. After CCND1 expression, the activity of the PI3K/Akt signaling pathway significantly increases [33]. Polygalacin D reduces the expression level of CDK2 protein, further slowing down the progression and metastatic risk of lung cancer A549 [34]. P53, as the most commonly mutated gene in cancer cells, is frequently involved in regulating the expression of genes related to cell proliferation and nuclear growth. P53 negatively regulates the expression of transcriptional activator 3 (STAT3), thereby exerting a significant inhibitory effect on cell proliferation, migration and invasion [35]. Increasing evidence suggests that mutated p53 plays an important role in the occurrence, invasion and migration of lung cancer [36, 37]. Such as Calvatia gigantea extract can reduce the expression of CCND1 and increase the expression of p53 in lung cancer A549 cells, further inducing cell cycle arrest and apoptosis [38, 39]. Sesamin can inhibit the expression of CDK2 and induce cell cycle arrest at the G1 phase and apoptosis in lung cancer A549 cells by regulating the Akt/p53 pathway. This further reduces the growth and metastasis of lung cancer [40]. AMPK is a downstream effector of SIRT1 and a key regulator of autophagy. Through the SIRT1/AMPK signaling pathway, it activates the formation of autophagosomes and increases the number of autophagolysosomes in lung cancer A549 cells, thereby promoting cell apoptosis [41]. FOXO proteins are involved in various cellular processes, including cell differentiation, cell apoptosis, cell proliferation, DNA damage, repair and functioning as mediators of oxidative stress [42]. Activation of the PI3K/AKT pathway leads to the phosphorylation and inactivation of FOXO transcription factors. This results in the downregulation of FOXO target genes involved in cell cycle arrest, apoptosis and cellular metabolism [43]. Such as miR-411 in lung cancer downregulates FOXO1, further influencing cell proliferation and cell survival in lung cancer [44]. The overexpression of lncFOXO1 suppresses the viability, colony formation and invasion of A549 cells, while increasing CCND1 expression, further activating the PI3K/AKT signaling pathway to exert anti-proliferative effects [45]. The findings from the aforementioned study are consistent with the results of our research, suggesting that the target proteins CDK2, CCND1, and E2F1 may serve as potential biomarkers and crucial therapeutic targets in lung cancer. The PI3K-Akt signaling pathway, p53 signaling pathway, FoxO signaling pathway and AMPK signaling pathway collectively regulate and influence the proliferation and survival of lung cancer cells.

To further validate the therapeutic effect of SMA on LC, molecular docking was performed to verify the interaction between the six alkaloid monomers of SMA and the target proteins 2W96, 2CCH, and 1O96. The results of molecular docking demonstrated that the six active components exhibited favorable binding affinity with the selected target proteins, forming the best complexes. In addition, the rationality of the results of network pharmacological screening was to further confirmed by cell experiments in vitro. This finding provides supporting evidence for the rationality of the network pharmacological screening results, suggesting the potential therapeutic efficacy of SMA in LC. The study provided a preliminary elucidation of the mechanism of SMA in treating lung cancer.

Although our study has identified potential biomarkers and key target proteins and signaling pathways for the treatment of lung cancer, there are still some limitations. Therefore, further cell-based experiments are necessary to elucidate the mechanisms of these targets and signaling pathways. For instance, we will further evaluate the impact of SMA on the proliferation of lung cancer cell lines H1299, H1975, PC9 and A549, and assess their therapeutic potential. Additionally, we will employ techniques such as Western blotting and molecular biology to validate key proteins and signaling pathways, and further explore the interactions between these pathways. Our subsequent experimental research will address these gaps and provide more evidence for the treatment of SMA on the LC.

## Conclusions

In this study, network pharmacology combined with molecular docking was used to elucidate the mechanism by which SMA regulate LC through multiple targets and channels. The results showed that oxymatrine, matrine, oxysophocarpine, sophocarpine, sophoridine and cytisine of SMA could form the best complex with 2W96, 2CCH and 1O96 target proteins, and to further verify the therapeutic effect of SMA in LC A549 cell, which also can provide a reliable reference basis that SMA were potential candidates in treating LC.

## Data Availability

The datasets used and analysed during the current study available from the corresponding author on reasonable request.
